# Impact of physical exercise on children with attention deficit hyperactivity disorders: Evidence through a meta-analysis

**DOI:** 10.1097/MD.0000000000017980

**Published:** 2019-11-15

**Authors:** Yu Zang

**Affiliations:** Department of Physical Education, Nanjing University, Nanjing, Jiangsu, PR China.

**Keywords:** anxiety, attention deficit hyperactivity disorders, confidence intervals, depression, impulsive symptoms, physical exercise, social problems, weight mean difference

## Abstract

**Background:**

Attention deficit hyperactivity disorder (ADHD) which is characterized by developmentally inappropriate levels of attention, hyperactivity and impulsivity, is considered as the most common neurodevelopmental disorder in childhood. Physical exercise has shown to have several benefits in the improvement of children with ADHD. In this meta-analysis, we aimed to systematically show, with evidence, the impact of physical exercise on children with ADHD.

**Methods:**

Web of Science, MEDLINE, EMBASE, Google Scholar, Cochrane Central and http://www.ClinicalTrials.gov were the searched sources for studies which were based on the impact of physical exercise on children with ADHD. Relevant endpoints were assessed. This evidence based meta-analysis was carried out by the most relevant RevMan 5.3 software. Due to the involvement of continuous data (mean and standard deviation), weight mean difference (WMD) with 95% confidence intervals (CI) were used to represent the final analysis. A significant level of *P* ≤ .05 was set and a fixed statistical effect model was used throughout the analysis.

**Results:**

Fourteen studies with a total number of 574 participants with ADHD were included in this evidenced based meta-analysis. Two hundred and seventy six (276) participants were assigned to the physical activity group whereas 298 participants were assigned to the control group. Results of this analysis showed that anxiety and depression were significantly improved with physical activity in these children with ADHD (WMD: -1.84; 95% CI: [-2.65 – (-1.03)], *P* = .00001). Hyperactive/impulsive symptoms (WMD: -0.01; 95% CI: [-0.32 – 0.29], *P* = .93) and inattention symptoms (WMD: -0.22; 95% CI: [-0.51 – 0.08], *P* = .15) were also improved with physical exercise but the results were not statistically significant. This evidence based analysis showed thought problems (WMD: -3.49; 95% CI: [-5.51 – (-1.47)], *P* = .0007), social problems (WMD: -5.08; 95% CI: [-7.34 – (-2.82)], *P* = .0001), and aggressive behaviors (WMD: -3.90; 95% CI: [-7.10 – (-0.70)], *P* = .02) to have significantly been improved in participants with ADHD who were assigned to physical activity group.

**Conclusions:**

This current meta-analysis showed with evidence, that physical exercise has a major contribution owing to significant improvement in anxiety and depression, aggressive behaviors, thought and social problems among children suffering from ADHD. Therefore, physical exercise should be incorporated in the daily life of children with ADHD. Further future research should be able to confirm this hypothesis.

## 1 Introduction

Attention deficit hyperactivity disorder (ADHD) which is characterized by developmentally inappropriate levels of attention, hyperactivity and impulsivity, is considered as one of the most common psychiatric disorders and as the most common neurodevelopmental disorder in childhood.^[1]^ ADHD cannot often be diagnosed through medical laboratory tests. The diagnosis is mainly based on parents’ and teachers’ reports, with reference to learning disabilities, anxiety and conduction disorders.^[2]^

Children with ADHD often have significant impairment in occupational,^[3]^ health,^[4]^ social^[5]^ and academic^[6]^ domains contributing to motivational and learning disabilities and anxiety disorders as well as impulsive behaviors resulting in a negative impact on themselves and therefore contribute to the development of suicidal ideas later during adulthood.^[7]^ A higher risk of academic failure, and poor psychological and occupational outcomes have strictly been related to ADHD.^[8]^ This disorder is also associated with substantial long-term depressive outcomes and mortality.^[9]^

To improve the symptoms of ADHD in these children, pharmacological treatment is associated with long-term beneficial effects and has been considered as first line therapy.^[10]^ However, the use of pharmacological drugs have potentially serious adverse events limiting their use in certain cases.^[11]^ Poor adherence,^[12]^ partial or non-response to medications,^[13]^ and uncertainty about the long-term costs of medications,^[14]^ beneficial and side effects are the other limitations of pharmacological therapy for ADHD.

Physical exercise has always been beneficial and is considered as a preventive measure for developing or the exacerbation of several chronic medical conditions, and disabilities.^[15]^ Several recent studies support the benefits of physical exercise on the cognitive performance and the management of behavioral symptoms in children with ADHD.^[16,17]^ However, there is limited evidence to show the effects of physical activities on childhood ADHD.

A systematic review and meta-analysis protocol which was published in the year 2019,^[18]^ would aim to show the effects of physical activity on executive function in children with ADHD. However, it was only a study protocol and there is still no information about when the final study will be published.

In this current meta-analysis, we aimed to systematically show, with evidence, the impact of physical exercise on children with ADHD.

## 2 Material and methods

### 2.1 Data sources and search strategies

Web of Science, MEDLINE, EMBASE, Google Scholar, Cochrane Central and http://www.ClinicalTrials.gov were the searched sources for studies which showed the impact of physical exercises on children with ADHD.

The searched terms included:

1. Attention deficit hyperactivity disorders;
2. Attention deficit hyperactivity disorders and physical exercise;
3. Attention deficit hyperactivity disorders and physical intervention;
4. Attention deficit hyperactivity disorders and exercise;
5. Attention deficit hyperactivity disorders and physical activity;
6. ADHD and physical exercise.

### 2.2 Criteria for inclusion

Criteria for inclusion consisted of studies:

(a) Which were randomized trials/observational cohorts (prospective or retrospective studies)/case-control studies which were published before July 2019;
(b) Showing the comparison of physical exercise versus a control group in children with ADHD;
(c) Reporting relevant endpoints based on the impact of physical activities on health improvement status of children with ADHD such as aggressive behaviors, perseverative errors, internalized and externalized problems, social and thought problems;
(d) Published in English language.

### 2.3 Criteria for exclusion

Criteria for exclusion consisted of studies:

(a) Showing the impact of physical exercise in children with ADHD without comparing it to any control group;
(b) Relevant endpoints based on health improvement status were not reported;
(c) Published in a different language;
(d) Literature reviews, systematic reviews and meta-analyses;
(e) Repeated studies.

### 2.4 Outcomes reported in the original studies

Table 1 lists the outcomes/endpoints observed in ADHD participants who were assigned to the physical activity versus the control group. Most of the common outcomes reported in the original studies were extracted and listed in Table 1.

**Table 1:**
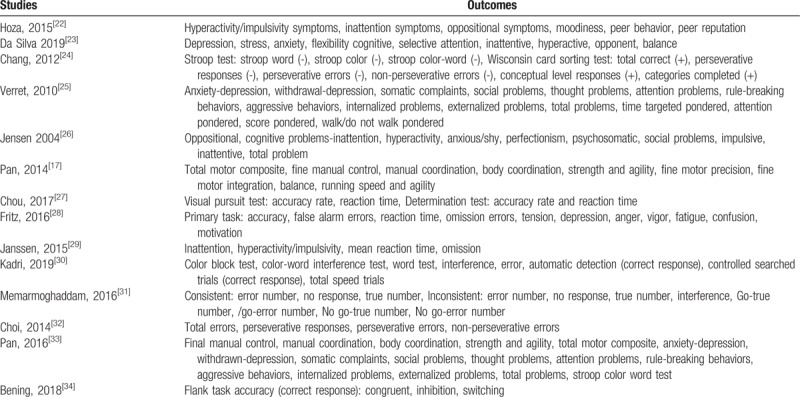
Outcomes reported in the original submissions.

### 2.5 Selected endpoints for this analysis

The endpoints which were assessed in this meta-analysis included:

1. Hyperactive/impulsive symptoms;
2. Anxiety and depression;
3. Inattention symptoms;
4. Oppositional symptoms;
5. Thought problems;
6. Social problems;
7. Aggressive behaviors;
8. Strength and agility;
9. Internalized problems;
10. Externalized problems;
11. Perseverative errors;
12. Non-perseverative errors;
13. Stroop color-word response.

### 2.6 Data extraction

The author extracted data including the common endpoints (associated with the improvement of health status of the participants) reported in the original studies consisting of the total number of participants in the physical and non-physical activity groups who suffered hyperactive/impulsive symptoms, anxiety and depression, inattention symptoms, oppositional symptoms, thought problems, social problems, aggressive behaviors, strength and agility, internalized and externalized problems, perseverative and non-perseverative errors, and stroop color-word response; data concerning the methodological quality of the studies, the total number of ADHD children assigned to the physical exercise and the control groups respectively, the baseline characteristics including the percentage of boys and girls with ADHD who were assigned to the physical activity versus the non-physical activity group respectively, the mean age which was reported in years and mean body mass index (BMI) of the participants which was reported in kilogram per meter square (Kg/m^2^), the mean and standard deviation values associated with each endpoints since these values would be required during data analysis, and the types of studies; that is, whether the study involved was a randomized trial, an observational cohort (retrospective or prospective) or a case-control study.

### 2.7 Quality assessment

The quality assessment of the randomized trials was carried out with reference to the criteria suggested by the Cochrane Collaboration^[19]^ and the quality assessment of the observational studies was carried out by the Newcastle Ottawa Scale (NOS).^[20]^

Based on the assessment, a grade was allotted to each study, grade ‘A’ indicating a low risk of bias, grade ‘B’ indicating a moderate risk and grade ‘C’ indicating a high risk of bias.

### 2.8 Statistical analysis

This evidence based meta-analysis was carried out by the most relevant RevMan 5.3 software. Due to the involvement of continuous data (mean and standard deviation), weight mean difference (WMD) with 95% confidence intervals (CI) were used to represent the final analysis. A significant level of *P* ≤ .05 was set and a fixed statistical effect model was used throughout the analysis. In addition to the *Q* statistic test with a *P* value ≤ .05 showing the level of significance, heterogeneity was also assessed by the I^2^ statistic test. The I^2^ value was reported in percentage. Heterogeneity was determined by an increasing I^2^ value. The higher the I^2^ value for a particular subgroup, the greater the heterogeneity for that particular subgroup.

During data extraction, a mean and standard deviation were provided from the parents’ as well as from the teachers’ perspective view. Therefore, in this analysis, we have calculated an average value of this mean and standard deviation to proceed with the results.

Sensitivity analysis was also calculated by an exclusion method. In addition, publication bias was observed through visual assessment of the funnel plot.

### 2.9 Ethical review

Ethical or board review approval was not required for this meta-analysis since data were extracted from previously published studies.

## 3 Results

### 3.1 Searched outcomes

A total number of 1456 publications were obtained from the above mentioned searched databases (PRISMA guidelines).^[21]^ An initial elimination of 1386 articles were due to irrelevance. Seventy (70) full text articles were assessed for eligibility based on the criteria for inclusion and exclusion.

Full text articles were eliminated since:

(a) They did not consist of control group (n = 15);
(b) They were literature reviews, systematic reviews and meta-analyses (n = 6);
(c) They did not report relevant outcomes (n = 6);
(d) They were case studies (n = 8);
(e) They were duplicated studies (n = 21).

**Figure 1:**
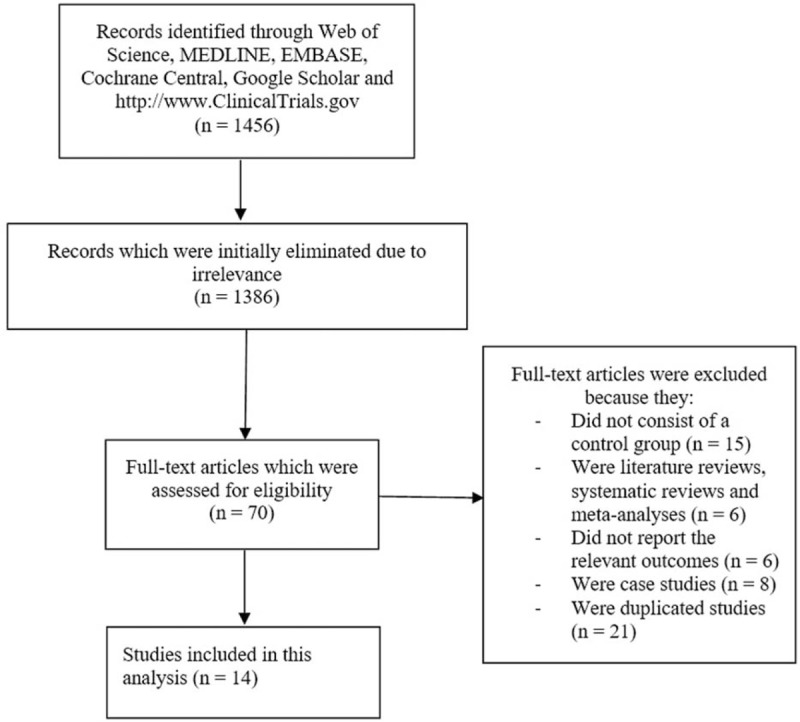
Flow diagram representing the study selection.

Finally, 14 studies^[17,22–26,27–34]^ were included in this evidence based meta-analysis, as shown in Figure 1.

### 3.2 Main and baseline features of the studies

A total number of 574 participants with ADHD were included in this evidenced based meta-analysis. Two hundred seventy six (276) participants with ADHD were assigned to the physical activity group whereas 298 participants with ADHD were assigned to the control group, as shown in Table 2.

**Table 2:**
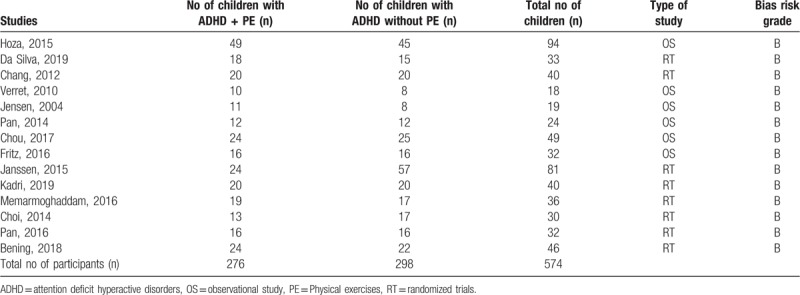
Main features of the selected studies.

Eight (8) studies were randomized trials, and 6 studies were observational studies.

Bias risk assessment of the randomized trials based on the Cochrane Collaboration, and the bias risk of the observational studies based on the NOS resulted in an average grade B score indicating a moderate risk of bias in each of the trial or study.

The baseline features including the total number of boys vs girls with ADHD, the mean body mass index of the participants, and the average age were listed in Table 3. One study was an exception and included adolescents with a mean age of 21 years. However, based on the major participants of this analysis, mean age of the children ranged from 8.29 to 16 years. The average percentage of boys and girls were (51% to 100%) and (10% to 49%) respectively.

**Table 3:**
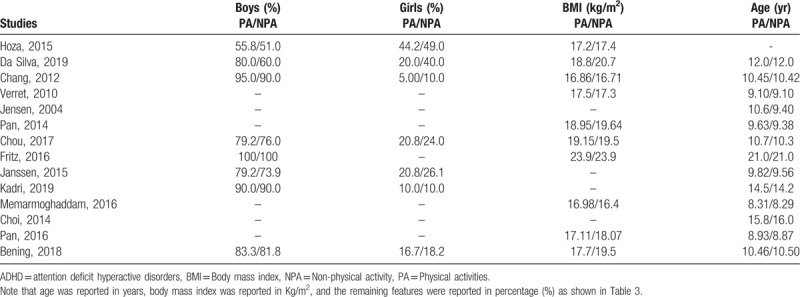
Baseline features of the children with ADHD.

### 3.3 Main results on the impact of physical exercise on children with ADHD

**Figure 2:**
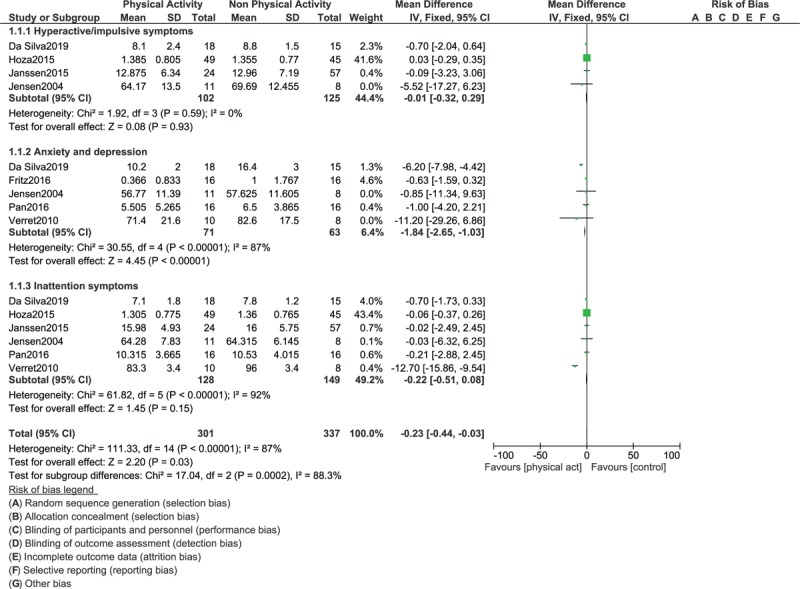
Comparing the endpoints in children with ADHD assigned to vs not assigned to physical exercise (Part I).

This evidence based meta-analysis showed that anxiety and depression were significantly improved with physical activity in these children with ADHD (WMD: -1.84; 95% CI: [-2.65 – (-1.03)], *P* = .00001) as shown in Figure 2. Hyperactive/impulsive symptoms (WMD: -0.01; 95% CI: [-0.32 – 0.29], *P* = .93) and inattention symptoms (WMD: -0.22; 95% CI: [-0.51 – 0.08], *P* = .15) were also improved with physical exercise, but the results were not statistically significant, as shown in Figure 2.

**Figure 3:**
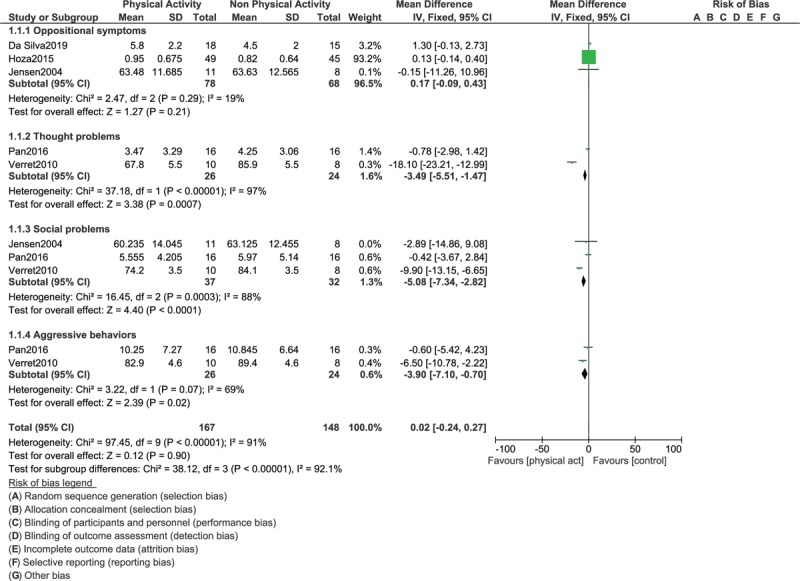
Comparing the endpoints in children with ADHD assigned to vs not assigned to physical exercise (Part II).

This evidence based analysis showed thought problems (WMD: -3.49; 95% CI: [-5.51 – (-1.47)], *P* = .0007), social problems (WMD: -5.08; 95% CI: [-7.34 – (-2.82)], *P* = .0001), and aggressive behaviors (WMD: -3.90; 95% CI: [-7.10 – (-0.70)], *P* = .02) to have significantly been improved in participants with ADHD who were assigned to physical activities as shown in Figure 3. Oppositional symptoms were not significantly improved (WMD: 0.17; 95% CI: [-0.09 – 0.43], *P* = .21).

**Figure 4:**
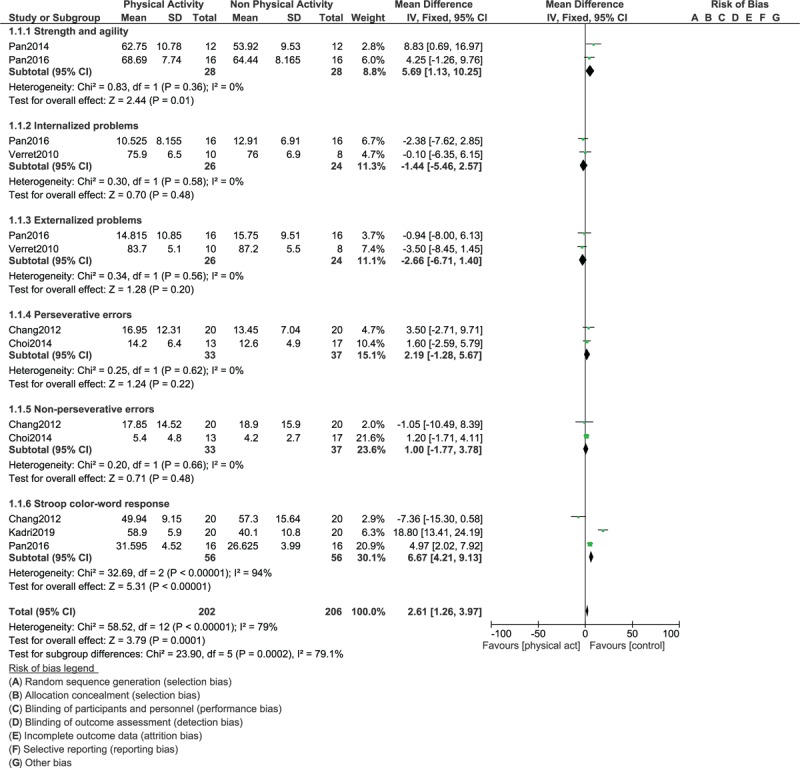
Comparing the endpoints in children with ADHD assigned to vs not assigned to physical exercise (Part III).

The results for strength and agility (WMD: 5.69; 95% CI: [1.13 – 10.25], *P* = .01), internalized problems (WMD: -1.44; 95% CI: [-5.46 – 2.57], *P* = .48), externalized problems (WMD: -2.66; 95% CI: [-6.71 – 1.40], *P* = .20), perseverative errors (WMD: 2.19; 95% CI: [-1.28 – 5.67], *P* = .22, non-perseverative errors (WMD: 1.00; 95% CI: [-1.77 – 3.78], *P* = .48) and stroop color-word test (WMD: 6.67; 95% CI: [4.21 – 9.13], *P* = .00001) as shown in Figure 4.

A summarized version of the results has been shown in Table 4.

**Table 4:**
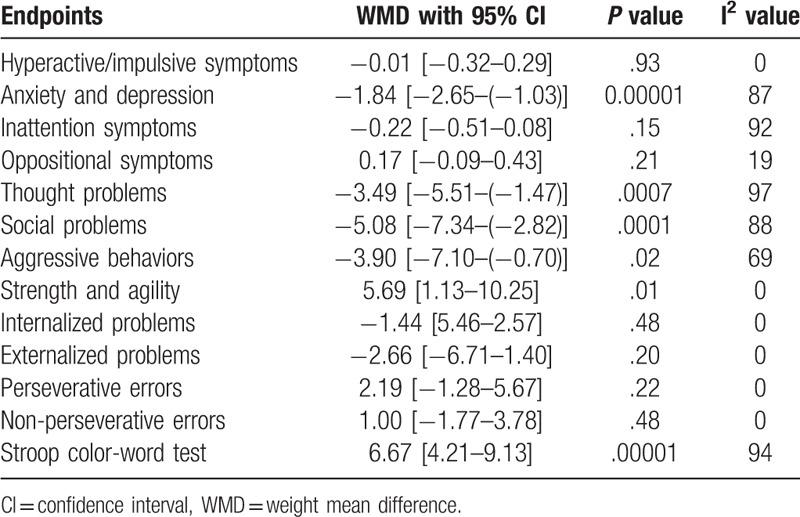
Summarized results.

**Figure 5:**
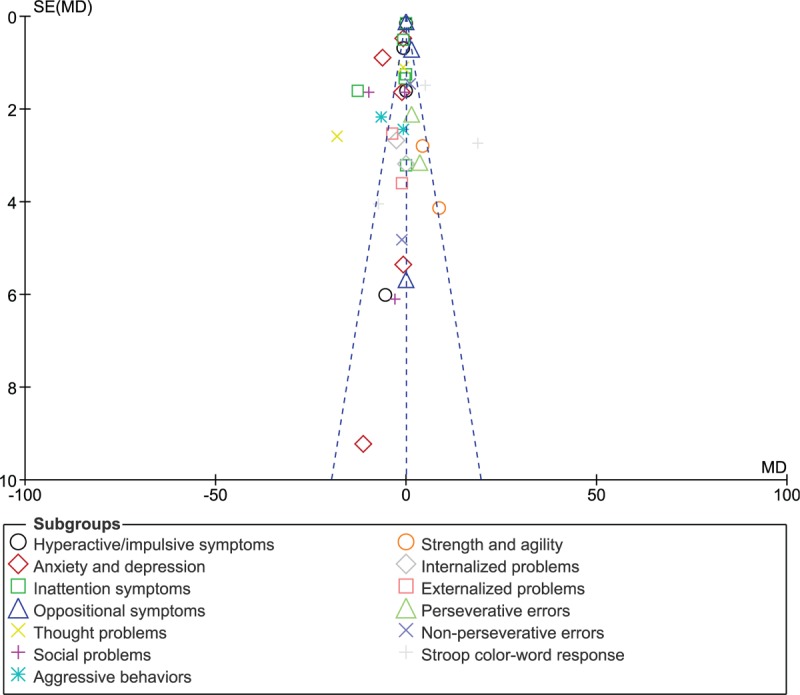
Funnel plot representing publication bias.

Consistent results were obtained throughout. Publication bias was visually observed through a funnel plot which has been demonstrated in Figure 5.

## 4 Discussion

Researchers have found intense exercise or physical activity to have major beneficial effects in children with ADHD.^[35]^ In our current meta-analysis, we aimed to show, with evidence, the benefits of physical exercise in children with ADHD.

Results of this analysis showed with evidence, that physical exercise significantly improved anxiety and depression, thought and social problems as well as any aggressive behavior. Hyperactive/impulsive as well as inattention symptoms were also improved, but without statistical significance.

Similarly, another meta-analysis involving 8 randomized trials based on aerobic and yoga exercises showed yoga and aerobic exercises to effectively improve hyperactivity, anxiety, impulsive disorders and social problems in children with ADHD.^[36]^ The authors stated that aerobic exercise had a major impact compared to yoga on these beneficial effects.

In a 6 week prospective trial, including 12 sessions of education and sports therapy whereby the participants were involved in a 90 minutes athletic activity twice a week, the authors demonstrated a strong so-relation of physical sports with improvement of symptoms of attention, cognitive and social skills in children with ADHD.^[37]^ A significant improvement in cognitive functions and social competency was noted in these children suggesting a major impact of physical exercises in children with ADHD. Similarly, another study showed physical activity to increase attention and calmness in a 4 year old ADHD boy.^[38]^ Another study has even demonstrated a possibly hypoactive attentional system in children with ADHD that could be induced and enhanced through engagement in physical exercise.^[39]^

Our current analysis showed that physical activities could reduce and improve anxiety and depression among children with ADHD. Even though this is a major benefit, other studies have shown that the duration of depression might also have an impact on the outcome. To be more clear on this matter, Ghio et al showed that untreated depression reported for a shorter time period was associated with better favorable outcomes and these patients responded well to the treatment given.^[40]^

Moreover, another review was also in favor of this current analysis.^[41]^ The authors stated that even if the study designs and the physical activities reported in the included studies varied considerably, all of them lead to similar conclusions indicating the beneficial effects of physical exercise in children with ADHD and led to significant improvements in terms of neuropsychological parameters, strength, motor skills and social behavior.

Therefore, with this positive impact of physical exercise in children with ADHD, it should be encouraged to improve the condition of children with ADHD in the society. Improvement in the academic field, ADHD children with healthier outcomes, can easily adapt and cope with normal children without ADHD, and would further contribute to the progress of a community, a country, or a continent. A society consisting of a subgroup of patients with ADHD but without depression, without suicidal attempts would further contribute to the well-being and progress of a country. It would be easier to set up better work environment later for these patients with ADHD.^[42]^ Parental stress will decrease and parental confidence in their children with ADHD will be increased.^[43]^

Social skill training is important in children with ADHD.^[44,45]^ Our current analysis showed physical exercise to significantly improve social problems in children with ADHD. Therefore, social training skills would also be improved without the requirement of significant assistance, and implication and patience of parents and teachers would be to a lower extent if these children become socially capable.

Depression is a major problem in children with ADHD. Scientific reports have shown pharmacological drugs which are used for the treatment of ADHD to further exacerbate depression in these subjects. This has been demonstrated in a case study involving a 7-year-old boy with ADHD who was on a minimum dose of methylphenidate. Increasing the dosage of the drug resulted in the development of clinical signs of depression. However, this unwanted condition was resolved when the drug was withdrawn.^[46]^ Therefore, improving depression through physical exercise by these children with ADHD will result in far less use of pharmacological drugs, thus sparing these children from adverse drug events representing another benefit of physical activity on ADHD.

## 5 Limitations

The limitations of this analysis were: first of all, only a total number of 574 participants were included. This minor number of participants might not lead to robust analysis. However, data were limited on this particular topic and there was no additional data that could be included. Secondly, the type of physical activity varied in this study. A few studies were based on swimming, basketball, and other physical training. However, since our analysis was based on physical exercise, all these activities were subsets of physical exercises. Thirdly, one study involved adolescents with a mean age of 21 years whereas all the other studies involved children. Another limitation could be the fact that three or four studies were not involved during data analysis since their endpoints were unique and were not reported in other studies for us to be able to make a comparison. Also, a high level of heterogeneity was observed in several of the subgroups. At last, only one researcher was involved in this study, and data extracted by only one researcher could result in bias, further limiting the strength of the results obtained.

## 6 Conclusions

This current meta-analysis showed with evidence, that physical exercise has a major contribution owing to significant improvement in anxiety and depression, aggressive behaviors, thought and social problems among children suffering from ADHD. Therefore, physical exercise should be incorporated in the daily life of children with ADHD. Further, future research should be able to confirm this hypothesis.

## Author contributions

**Conceptualization:** Yu Zang.

**Data curation:** Yu Zang.

**Formal analysis:** Yu Zang.

**Funding acquisition:** Yu Zang.

**Investigation:** Yu Zang.

**Methodology:** Yu Zang.

**Project administration:** Yu Zang.

**Resources:** Yu Zang.

**Software:** Yu Zang.

**Supervision:** Yu Zang.

**Validation:** Yu Zang.

**Visualization:** Yu Zang.

**Writing – original draft:** Yu Zang.

**Writing – review & editing:** Yu Zang.
